# Estimated glomerular filtration rate in post COVID-19 patients at 3–6 months and 12–18 months after infection

**DOI:** 10.1080/0886022X.2025.2551737

**Published:** 2025-09-02

**Authors:** Merel E. B. Cornelissen, Lizan D. Bloemsma, Nadia Baalbaki, Jos W. R. Twisk, George S. Downward, Anke-Hilse Maitland-van der Zee, on behalf of the P4O2 consortium

**Affiliations:** ^a^ Department of Pulmonary Medicine, Amsterdam UMC, University of Amsterdam, Amsterdam, The Netherlands; ^b^ Amsterdam Institute for Infection and Immunity, Amsterdam, The Netherlands; ^c^ Amsterdam Public Health, Amsterdam, The Netherlands; ^d^ Department of Epidemiology and Data Science, Amsterdam University Medical Centers, Amsterdam, The Netherlands; ^e^ Department of Environmental Epidemiology, Institute for Risk Assessment Sciences (IRAS), Utrecht University, Utrecht, The Netherlands; ^f^ Department of Global Public Health and Bioethics, Julius Center for Health Sciences, University Medical Center Utrecht, The Netherlands; ^g^ Department of Genetics, University Medical Center Groningen, The Netherlands

**Keywords:** Post COVID-19, estimated glomerular filtration rate, kidney function, renal impairment

## Abstract

**Background:**

Some degree of renal impairment is common during acute COVID-19 infection. However, it remains unclear whether this impairment is temporary or persists long term. In this study we compare kidney function (*via* estimated glomerular filtration rate [eGFR]) during infection, 3–6 months and 12–18 months after infection; the relationship between patient characteristics and eGFR in post COVID-19 patients; and the difference in eGFR between post COVID-19 patients and controls.

**Methods:**

In total, 95 post COVID-19 patients and 94 controls were included. Post COVID-19 patients were seen 3–6 months and 12–18 months after infection for biological sample collection and questionnaire administration, with results for biological samples during acute infection sourced from medical records. Mixed model analyses were performed to study the associations between patient characteristics and eGFR and linear regression analyses to study the difference between post COVID-19 patients and controls.

**Results:**

Under a complete case analysis among post COVID-19 patients (where results available at the acute phase and both follow-up points, *n* = 61), the eGFR was <90 mL/min/1.73 m^2^ in 50.8% during infection, in 68.9% at visit 1 and in 75.4% at visit 2, compared with 40.4% in the control group. The eGFR was lower among patients with a higher age, those who had been hospitalized, and those with CVD/hypertension. After adjusting for confounders, the eGFR at the 12–18 month time point was significantly lower in post COVID-19 patients than controls.

**Conclusions:**

Previous COVID-19 infection was associated with a reduced eGFR up to 18 months after infection with higher age and CVD/hypertension increasing this likelihood.

## Introduction

While the acute coronavirus disease 2019 (COVID-19) pandemic is less of a global concern than several years ago, post COVID-19 condition remains a public health problem. It is estimated that 10-30% of those who had COVID-19 suffer from this condition [1–3], which is defined as symptoms that are newly developed or still present at three months after acute infection, last for at least two months and cannot be explained by an alternative diagnosis [4].

Recent studies suggest that COVID-19 can lead to renal impairment, likely *via* the virus’ spike proteins binding to the angiotensin-converting enzyme receptor 2 (ACE2) on the surface of kidney cells, allowing the virus to fuze with the host cells and contribute to acute kidney injury (AKI) [5–7]. Several studies have reported AKI (defined as an increase in creatinine of ≥50% or a decrease in estimated glomerular filtration rate (eGFR) of ≥25% [8]) in approximately 10-20% of patients hospitalized with COVID-19 [9]. Another study reported a mild renal impairment (defined as an eGFR of <90 mL/min/1.73 m^2^) in approximately 50% of COVID-19 patients after 12 months, with one in seven having a more severe impairment (<60 mL/min/1.73 m^2^) [10]. It has also been reported that, over 12 months, renal function declined by approximately 3.4% (2.96 mL/min/1.73 m^2^) with those who were hospitalized having the largest reduction (6.7%). Several risk factors for impaired renal function in relation to COVID-19 have been identified, including sex, COVID-19 severity, and comorbidities [11]. COVID-19 patients who develop AKI have significantly worse outcomes, may develop chronic kidney disease (CKD), require dialysis [9], or die prematurely [12]. For example, in a group of COVID-19 patients admitted to the ICU, 33.3% of the patients with AKI died, compared with 8.9% of the non-AKI patients [13].

It is still largely unknown whether the renal impairment observed in post COVID-19 patients is temporary or whether it persists long term. Therefore, the aim of this study is to examine kidney function (*via* eGFR) during the acute phase, at 3–6 months and at 12–18 months after infection in the P4O2 COVID-19 cohort. The second aim is to study the associations between patient characteristics and eGFR in post COVID-19 patients. The third aim is to compare eGFR between post COVID-19 patients and controls (i.e., a group of participants without post COVID-19).

## Materials and methods

### Study design and population

#### Post COVID-19 patients

The P4O2 COVID-19 study is a multicenter, prospective, observational cohort study in the Netherlands. Details of the study design have been described by Baalbaki et al. [14]. In brief, 95 ex-COVID-19 patients were recruited between May 2021 and September 2022 at post COVID-19 outpatient clinics in five Dutch hospitals: Amsterdam UMC (location AMC and VUmc), Leiden UMC, Spaarne Gasthuis and VieCuri. Patients visited the post COVID-19 outpatient clinic 3–6 months after hospitalization or, if not hospitalized, were referred to the outpatient clinic by their general practitioner three months after their positive polymerase chain reaction (PCR) test. Inclusion criteria were a confirmed SARS-CoV-2 infection (by PCR, serology tests or a COVID-19 Reporting and Data System (CO-RADS) score 4 or 5), the ability to provide informed consent, being aged 40-65 years, access to internet and understanding of the Dutch language. Exclusion criteria were the inability to provide informed consent, a terminal illness or participation in another study involving investigational or marketed products either concurrently or within four weeks prior to study entry. All participants were invited for two study visits: at 3–6 and 12–18 months after hospitalization or positive PCR test.

#### Control group

Participants of the P4O2 PARASOL cohort (Prevention of And Risk fActorS for chronic diseases: an Observational study in North HoLland) are included in the current study as a control population. Recruitment to the PARASOL cohort began in March 2024 and is ongoing, with participants being recruited from the municipalities of Amsterdam and Hoorn. Currently, 112 participants are included. Inclusion criteria were aged 40-55 years, the ability to provide informed consent and understanding of the Dutch language. Exclusion criteria were the inability to provide informed consent or having a serious mental impairment, i.e., not able to understand the study protocol. Furthermore, for the current study, participants who reported having post COVID-19 symptoms, as well as those who did not have an eGFR measured were excluded.

#### Ethical approval

Ethical approval for the P4O2 Covid-19 study was provided by the ethical board of the Amsterdam University Medical Center (UMC), reference number NL74701.018.20. Ethical approval for the P4O2 PARASOL cohort was provided by the ethical board of the Amsterdam University Medical Center (UMC), reference number NL84012.018.23. Written informed consent was gained from all participants and participants could freely withdraw from the study at any time for any reason.

### Data collection

For the COVID-19 cohort, at the first study visit (*t* = 1), written informed consent was obtained, blood was collected and questionnaires were administered. Furthermore, baseline characteristics concerning the patient’s health status prior to and during COVID-19 were obtained from electronic patient files and cardiovascular disease (CVD) and hypertension were determined with a questionnaire where patients reported if they were ever diagnosed with CVD and/or hypertension. Renal function was calculated *via* eGFR, based on creatinine levels in the collected blood samples using the formula CKD-EPI [15,16]:

GFR = 142 × min (C/K or 1)^α^ × max (C/K or 1)^−1.200^ × 0.9938^Age^ (× 1.012 for females)

C = serum creatinine in mg/dL

*K* = 0.7 (females) and 0.9 (males)

α = −0.241 (females) and −0.302 (males)

For those who were hospitalized, the eGFR during hospitalization was also calculated (*t* = 0). A second study visit (*t* = 2) was executed 9–12 months later, and the same measurements were performed.

For the PARASOL cohort, written informed consent was obtained during the study visit and the same measurements were performed as in the COVID-19 cohort. CVD was determined with a questionnaire where participants reported if they were ever diagnosed with CVD. Hypertension was based on blood pressure measurements, defined as systolic pressure >140 mmHg and diastolic pressure >90 mmHg.

### Data analysis

The mean eGFR was calculated for the post COVID-19 patients at all three timepoints and at the time of inclusion for the control group. Changes in eGFR between timepoints were also calculated for the post COVID-19 patients. To better identify trends over time, analysis was also repeated for ‘complete cases’, i.e., those who contributed blood samples at *t* = 0, *t* = 1, and *t* = 2. To study the associations between patient characteristics and eGFR in post COVID-19 patients, univariable linear mixed models were developed, including all three timepoints with a random intercept on patient level. In these models, eGFR was used as the outcome and the patient characteristics as determinants. Due to the small number of participants per group, binary variables were created for each categorical determinant: ethnicity (Caucasian vs. other), level of education (university or higher professional education (high) vs. no education or primary or secondary education (low)), and smoking status (current or ex smoker vs. never smoker). All determinants with *p* < 0.1 were included in a multivariable linear mixed model. Lastly, linear regression analyses were conducted to compare eGFR at *t* = 1 and *t* = 2 in the post COVID-19 group with the control group. A Directed Acyclic Graph (DAG) [17] was created to explore the potential confounders for the comparison between the post COVID-19 group and the control group (Figure S1).

Statistical analyses were performed using R software version 4.2.1.

#### Sensitivity analyses

To examine the role of other contributing factors such as prior covid infection, age, and comorbidity on findings, four sensitivity analyses were performed. First, to more directly compare differences in renal function between those with and without post COVID-19 but have also been infected with COVID-19, analysis was repeated retaining only controls who reported having had a positive COVID-19 test. Second, to evaluate any potential role of cardiovascular drivers of kidney disease, analysis was performed restricted only to those without CVD or hypertension. Third, to evaluate the wider role of comorbidities, this was expanded to exclude participants with any prior comorbidities (CVD/hypertension, diabetes, auto-immune disease, or pulmonary disease). Finally, as the control group had a mean age several years younger than that of the post COVID-19 group, patients in the post COVID-19 group aged >55 years were excluded in order to make the two groups more comparable.

## Results

### Baseline characteristics

In total, 95 post COVID-19 patients and 94 controls were included in this study. For the control group, participants who had post COVID-19 symptoms (or had a missing value) (*n* = 15) or a missing eGFR value (*n* = 3) were excluded. Table 1 shows the baseline characteristics of both groups. The mean age of the post COVID-19 patients was 54.2 years, with 50.5% being male. Of these patients, 89.5% were hospitalized and 28.7% were admitted to the intensive care unit (ICU). When examining comorbidities, 35.8% had CVD or hypertension, 15.8% had diabetes, 7.4% had an auto-immune disease and 22.1% had a pulmonary disease (COPD, asthma and/or ILD). The mean age of the control group was 48.6 years and 53.2% were male. When examining comorbidities, 4.3% had CVD or hypertension, 1.1% had diabetes, 5.5% had an auto-immune disease and 4.3% had a pulmonary disease.

**Table 1.** Baseline characteristics of the post COVID-19 patients and controls.

	Post COVID-19 patients (*n* = 95)	Controls (*n* = 94)
Age in years	54.2 ± 6.2	48.6 ± 4.8
Sex		
Male	48 (50.5)	50 (53.2)
Female	47 (49.5)	44 (46.8)
BMI in kg/m^2^	30.5 ± 5.3 (*n* = 94)	25.9 ± 4.5
Ethnicity		
Caucasian	67/87 (77.0)	71/90 (78.9)
Other	20/87 (23.0)	19/90 (21.1)
Level of education		
No education completed	NA	1/92 (1.1)
Primary and secondary education	52/79 (65.8)	43/92 (46.7)
Higher professional education	18/79 (22.8)	22/92 (23.9)
University education	9/79 (11.4)	26/92 (28.3)
Smoking status		
Current	4 (4.2)	10/93 (10.8)
Ex	51 (53.7)	30/93 (32.3)
Never	40 (42.1)	53/93 (57.0)
Comorbidities		
CVD/hypertension	34 (35.8)	4 (4.3)
Diabetes	15/94 (16.0)	1 (1.1)
Auto-immune disease	7 (7.4)	5/90 (5.6)
Pulmonary disease*	21/94 (22.3)	4/89 (4.5)
Acute COVID-19 severity		
Mild	10 (10.5)	NA
Moderate	61 (64.2)	NA
Severe	24 (25.3)	NA
Hospitalized	85 (89.5)	NA
Admitted to ICU	27/94 (28.7)	NA

* Includes Chronic Obstructive Pulmonary Disease (COPD), asthma and interstitial lung disease (ILD).

Values are shown as mean ± SD or n (%). BMI: body mass index, CVD: cardiovascular disease, ICU: intensive care unit.

### Estimated glomerular filtration rate

The eGFR tended to be lower among post COVID-19 patients than in controls, especially among the sub-group who provided samples at all three time points. Specifically, among post COVID-19 patients, at *t* = 0 (*n* = 84, only measured in hospitalized patients) the mean eGFR was 85.3 mL/min/1.73 m^2^, with 45.2% having an eGFR <90 and 11.9% having an eGFR <60. At *t* = 1 (*n* = 95), the mean eGFR was 84.7 mL/min/1.73 m^2^, with 54.7% having an eGFR <90 and 8.5% having an eGFR <60. At *t* = 2 (*n* = 68), the mean eGFR was 80.9 mL/min/1.73 m^2^, with 70.6% having an eGFR <90 and 5.9% having an eGFR <60. The mean eGFR in the control group was 91.2 mL/min/1.73 m^2^, 40.4% had an eGFR < 90 and 1.1% had an eGFR < 60 (Table 2).

**Table 2.** eGFR in Post COVID-19 patients per timepoint and in controls.

	COVID-19 cohort						PARASOL cohort
	Acute phase		Visit 1		Visit 2		
	All participants (*n* = 84)	Complete case (*n* = 61)^a^	All participants (*n* = 95)	Complete case (*n* = 61)^a^	All participants (*n* = 68)	Complete case (*n* = 61)^a^	Controls (*n* = 94)
eGFR (mL/min/1.73 m^2^)	85.3 ± 21.6	82.3 ± 21.3	84.7 ± 16.5	80.7 ± 16.4	80.9 ± 16.6	79.4 ± 16.1	91.2 ± 15.2
eGFR < 90 mL/min/1.73 m^2^	38 (45.2)	31 (50.8)	52 (54.7)	42 (68.9)	48 (70.6)	46 (75.4)	38 (40.4)
eGFR < 60 mL/min/1.73 m^2^	10 (11.9)	8 (13.1)	8 (8.5)	6 (9.8)	4 (5.9)	4 (6.6)	1 (1.1)
Difference in eGFR*	NA	NA	−1.5 ± 16.7	−1.6 ± 16.6	−1.3 ± 9.2	−1.3 ± 8.9	NA
Decrease in eGFR*	NA	NA	50/84 (59.5)	36 (59.0)	37/68 (54.4)	34 (55.7)	NA

Values are shown as mean ± SD or n (%). eGFR: estimated Glomerular Filtration Rate.

* Only measured in patients who had a measurement at both the current and previous timepoints.

^a^ Complete case: Participants who provided a blood sample during the acute phase, at visit 1, and visit 2.

From acute phase to visit 1 the eGFR decreased by an average of 1.5 mL/min/1.73 m^2^ and from visit 1 to visit 2 this decrease was 1.3 mL/min/1.73 m^2^. From acute phase to visit 2 the eGFR decreased with 2.93 mL/min/1.73 m^2^.

When restricting to post COVID-19 patients who provided eGFR measurements at all three timepoints (*n* = 61), the mean eGFR at *t* = 0 was 82.3 mL/min/1.7 3m^2^, with 50.8% having an eGFR <90 and 13.1% having an eGFR <60. At *t* = 1, the mean eGFR was 80.7 mL/min/1.73 m^2^, with 68.9% having an eGFR <90 and 9.8% having an eGFR <60. At *t* = 2, the mean eGFR was 79.4 mL/min/1.73 m^2^, with 75.4% having an eGFR <90 and 6.6% having an eGFR <60 (Table 2).

### Mixed models for post COVID-19 patients

In univariable mixed model analyses, the eGFR was lower for those with a higher age (coefficient [95% CI]: −0.72 [−1.25, −0.19] per year increase), hospitalized patients (−10.71 [−21.90, 0.48]) and patients with CVD or hypertension (−8.16 [−14.98, −1.34]. However, when combining these three determinants in one model, the relationships, while still directionally the same as the univariable models, were no longer statistically significant (Table 3).

**Table 3.** Results of univariable and multivariable mixed model analyses examining determinants of eGFR in post COVID-19 patients.

	Estimate (95%CI)	
	Univariable	Multivariable
Age in years	**−0.72 (−1.25, −0.19)**	−0.51 (−1.06, 0.05)
Sex (male)	−3.64 (−10.33, 3.06)	
BMI in kg/m^2^	−0.21 (−0.84, 0.43)	
Ethnicity (Caucasian)	2.37 (−4.64, 9.37)	
Level of education (high)	1.40 (−6.43, 9.22)	
Smoking (ever)	−1.11 (−5.70, 7.93)	
Hospitalized	−10.71 (−21.90, 0.47)	−6.66 (−17.88, 4.57)
ICU	−1.28 (−8.77, 6.22)	
CVD/hypertension	**−8.16 (−14.98, −1.34)**	−5.95 (−12.83, 0.93)
Diabetes	−0.76 (−9.98, 8.47)	
Pulmonary disease	−0.32 (−8.45, 7.80)	

BMI: body mass index, CVD: cardiovascular disease, CI: confidence interval, eGFR: estimated glomerular filtration rate, ICU: intensive care unit. Determinants with *p* < 0.1 are included in multivariable model. Statistically significant results are highlighted in bold (*p* < 0.05).

### Comparison between post COVID-19 and control group

In linear regression models comparing eGFR at visit 1 or visit 2 against controls, being in the post COVID-19 group was significantly associated with a reduced eGFR in univariable analyses (Table 4). For visit 1, the eGFR among those in the post COVID-19 group was 6.45 mL/min/1.73 m^2^ lower than the control group (95% CI: −11.01, −1.90) and for visit 2, the eGFR was 10.26 mL/min/1.73 m^2^ lower (95% CI: −15.23, −5.28). After adjusting for potential confounders (age, sex, BMI, smoking status, low socioeconomic status (i.e., level of education), CVD/hypertension and diabetes), for visit 1 the effect was no longer statistically significant (estimate [95%CI]: −5.12 [−11.97, 1.72]), but for visit 2 the effect remained significant (−7.50 [−14.37, −0.62]).

**Table 4.** Results of linear regression analyses examining differences in eGFR between post COVID-19 patients (at *t* = 1 and *t* = 2) and controls.

	Estimate (95% CI)	
	Unadjusted	Adjusted*
Post COVID-19 (*t* = 1)	**−6.45 (−11.01, −1.90)**	−5.12 (−11.97, 1.72)
Post COVID-19 (*t* = 2)	**−10.26 (−15.23, −5.28)**	**−7.50 (−14.37, −0.62)**

^*^ Adjusted for age, sex, BMI, smoking, level education, CVD/hypertension, and diabetes.

BMI: body mass index, CVD: cardiovascular disease, CI: confidence interval. Statistically significant results are highlighted in bold (*p* < 0.05).

The findings of the sensitivity analysis were generally consistent with those in the main analysis, finding reduced eGFR among those with post COVID-19 compared to the control population. In the first sensitivity analysis (where controls with no history of COVID-19 were excluded), the adjusted effect was significantly different (estimate [95%CI]: −7.69 [−15.07, −0.31]) at *t* = 1 (Table S1). In the second (those with CVD/hypertension excluded) and third (those with any comorbidities excluded) sensitivity analyses, the adjusted effects showed significant reductions in eGFR at *t* = 2 (Table S2 and S3). The only exception to these findings was the fourth sensitivity analyses (exclusion of those >55 years), where none of the adjusted models showed a significant association between post COVID-19 status and eGFR, although there was a negative association between eGFR and post COVID-19 status at *t* = 2 (estimate: −5.16 [−12.04, 1.72], Table S4).

## Discussion

The current study aimed to examine the impact of COVID-19 on renal function, finding a reduced eGFR in post COVID-19 patients from the acute phase up until 12–18 months after infection. Within this group, the eGFR was lower for older participants, those who were hospitalized or those with a history of CVD or hypertension. The eGFR was higher in the control group compared to the post COVID-19 patients. This difference appeared even larger 12–18 months after COVID-19.

These findings are consistent with those published elsewhere where a reduced eGFR has been reported at 12 months or more following COVID-19 [10,18]. In a study of 2,212 patients, who had long-COVID, Atiquzzaman et al. [10] reported a decrease in eGFR of 2.96 mL/min/1.73 m^2^ within one year after infection, consistent with the 2.93 mL/min/1.73 m^2^ decrease at 12–18 months after infection reported in the current study. Furthermore, they reported that 40% were at moderate to high risk of developing CKD based on the Kidney Disease: Improving Global Outcomes (KDIGO) 2012 prognosis guidelines. Their study population was broadly comparable to that within the present paper (for example, mean age of 56 versus 54 here and 51% male 51% here) with the exception of diabetes, which had a prevalence of approximately twice of that in the current population (35% versus 16%). Bento et al. [18] reported a reduction from 95.9 mL/min/1.73 m^2^ at infection to 65.9 mL/min/1.73 m^2^ at 16 months after infection, a larger reduction than reported in the current study. However, they included patients who developed AKI after COVID-19, compared to our inclusion of post COVID-19 patients regardless of whether they had AKI, which likely explains why they observed a more marked reduction than that reported here.

Similarly, when comparing to a control population, Schmidt-Lauber et al. [19] reported that COVID-19 patients had a 1.84 mL/min/1.73 m^2^ lower eGFR than controls at nine months after infection. They used a non-COVID-19 control group, matched on age, sex and education. However, while they found a slightly lower eGFR in COVID-19 cases, they found no evidence for ongoing kidney disease after mild or moderate COVID-19. Boruga et al. [20] used COVID-19 cases who did not develop post COVID-19 as a control group, reporting a lower eGFR in post COVID-19 patients compared to the control group at six months after infection (65.3 mL/min/1.73 m^2^ vs. 91.2 mL/min/1.73 m^2^).

Age is a well known risk factor for acute and chronic kidney injury, with a recent meta-analysis reporting that kidney function recovery after AKI is impaired in individuals aged > 65 years [21]. This appears to extend to renal impairment following COVID-19 with recent studies reporting that reduced eGFR due to COVID-19 was associated with higher age [18,22], consistent with the findings reported here. Being hospitalized has also been reported as a risk factor for reduced eGFR in post COVID-19 patients [10,23], which is consistent with our observations.

It is also well known that diabetes is a risk factor for kidney disease [24,25], but its interaction with COVID-19 remains unclear. Guzman-Esquivel et al. [23] reported a protective effect of having diabetes, where patients with diabetes had a higher eGFR after COVID-19 compared to non-diabetic patients. Conversely, Atiquzzaman et al. [10] reported a larger decline in eGFR for diabetic patients. The findings of the current study do not align with either study, as we found no effect of diabetes on eGFR in the post COVID-19 group. These differences could be due to differences in population selection. The study of Guzman-Esquivel et al. only included unvaccinated patients whereas Atiquzzaman et al. included patients aged ≥18 years and had a higher prevalence of diabetic patients. In the current study, we included patients aged between 40 and 65 years, regardless of vaccination status.

While CVD can be a risk factor for kidney disease (and vice-versa) [26,27], no other study, to our knowledge, has investigated the association between CVD and eGFR in post COVID-19 patients. However, Mirijello et al. [22] reported that the percentage of patients with hypertension was higher in post COVID-19 patients with eGFR <60 mL/min/1.73 m^2^ compared to eGFR ≥60 mL/min/1.73 m^2^ (66.7% vs. 39.9%).

### Strengths and limitations

The current study has several strengths, including a well-defined cohort and the use of a control group, which allowed us to identify a difference in eGFR between having post COVID-19 and those who do not. However, as the control group was both younger and healthier (i.e., fewer comorbidities), these findings must be interpreted with caution. In an effort to address this limitation we performed several sensitivity analyses in order to make the control and post COVID-19 groups more comparable, typically finding consistent results across these tests (except for restricting by age), providing support for the current findings.

Despite its strengths, the current study also has some limitations. First, no information on pre-COVID renal function was available, limiting our ability to conclude whether the impaired renal function observed here was primarily due to COVID-19 or preexisting infection (although we note similar findings after removing those with co-morbidities). Similarly, we were unable to evaluate whether participants were reviewed by nephrologists during their hospital admission, nor whether they received any post-discharge nephrological care. However, in a study by Guzman-Esquivel et al. [23], who did compare pre-COVID renal function, it was reported that 30% of the patients with a normal eGFR (≥90 mL/min/1.73 m^2^) pre-COVID had a reduced eGFR one year after infection, indicating that a low eGFR could be caused by COVID-19. A second limitation is the relatively small sample size, limiting statistical power. When performing the regression analyses, some groups contained only a few persons, therefore leading to higher uncertainty. Third, eGFR was the only metric utilized to examine kidney function, with no information on urine content (e.g., proteinuria) or histology being available, limiting a wider evaluation of the clinical impact of the observed findings. Fourth, the control group used in the current study was somewhat different from the post COVID-19 group, both in terms of age and comorbidities, limiting the extent to which these groups can be compared. However, in a series of sensitivity analyses, designed to improve comparability between these groups, consistent overall findings were found with those of the main analysis.

## Conclusion

The current paper reports a reduced eGFR up to 18-months following COVID-19 which was also associated with a higher age and CVD/hypertension. Furthermore, a difference in eGFR between post COVID-19 patients and controls was observed. These results suggest that, among patients who are developing (or who have developed) post COVID-19 condition, renal function should be monitored. Future research should focus on longer term outcomes, including whether the observed renal impairment leads to any clinical manifestation. Furthermore, to fully assess kidney function, additional variables like proteinuria and albumin levels should be considered.

## Supplementary Material

Renal_Failure_long_COVID_Fig_S1_DAG.jpeg

Supplementary materials_resubmission.docx

## Data Availability

The data underlying this article are not publicly available due to agreements made by the consortium, which only allow access by each consortium partner to specific data that answers their pre-specified research questions, but they are available from the corresponding author on reasonable request. A request for access to the data by organizations outside of the consortium can be submitted to the P4O2 Data Committee (*via* p4o2@amsterdamumc.nl) and the research will need to be performed in collaboration with one of the P4O2 consortium partners.
