# Supplementary figures and images for: Estimated glomerular filtration rate in post COVID-19 patients at 3–6 months and 12–18 months after infection
Source: Ren Fail. 2025 Sep 2;47(1):2551737. doi: 10.1080/0886022X.2025.2551737 (PMC12406321; doi:10.1080/0886022X.2025.2551737)

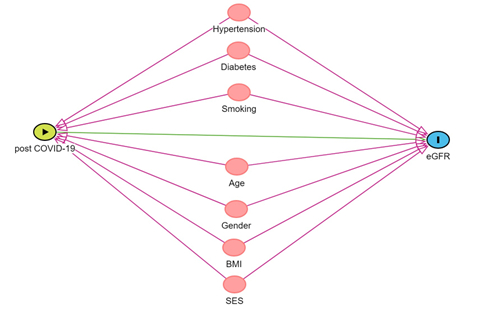

Supplement: Renal_Failure_long_COVID_Fig_S1_DAG.jpeg [file IRNF_A_2551737_SM3341.jpeg]
