# Supplementary material for: Estimated glomerular filtration rate in post COVID-19 patients at 3–6 months and 12–18 months after infection
Source: Ren Fail. 2025 Sep 2;47(1):2551737. doi: 10.1080/0886022X.2025.2551737 (PMC12406321; doi:10.1080/0886022X.2025.2551737)
Supplement: Supplementary materials_resubmission.docx [file IRNF_A_2551737_SM3340.docx]

Supplementary materials: Estimated Glomerular Filtration Rate in post COVID-19 patients at 3-6 months and 12-18 months after infection

Merel E.B. Cornelissen^1,2,3^, Lizan D. Bloemsma^1,2,3^, Nadia Baalbaki^1,2,3^, Jos W.R. Twisk^4^, George S. Downward^5,6^*, Anke H. Maitland-van der Zee^1,2,3,7^* on behalf of the P4O2 consortium

*shared last author

1. Department of Pulmonary Medicine, Amsterdam UMC, University of Amsterdam, Amsterdam, The Netherlands
2. Amsterdam Institute for Infection and Immunity, Amsterdam, The Netherlands
3. Amsterdam Public Health, Amsterdam, The Netherlands
4. Department of Epidemiology and Data Science, Amsterdam University Medical Centers, Amsterdam, The Netherlands
5. Department of Environmental Epidemiology, Institute for Risk Assessment Sciences (IRAS), Utrecht University, Utrecht, The Netherlands
6. Department of Global Public Health and Bioethics, Julius Center for Health Sciences,University Medical Center Utrecht, The Netherlands
7. Department of Genetics, University Medical Center Groningen, The Netherlands

# Corresponding author

G.S. Downward [g.s.downward@uu.nl](mailto:g.s.downward@uu.nl)


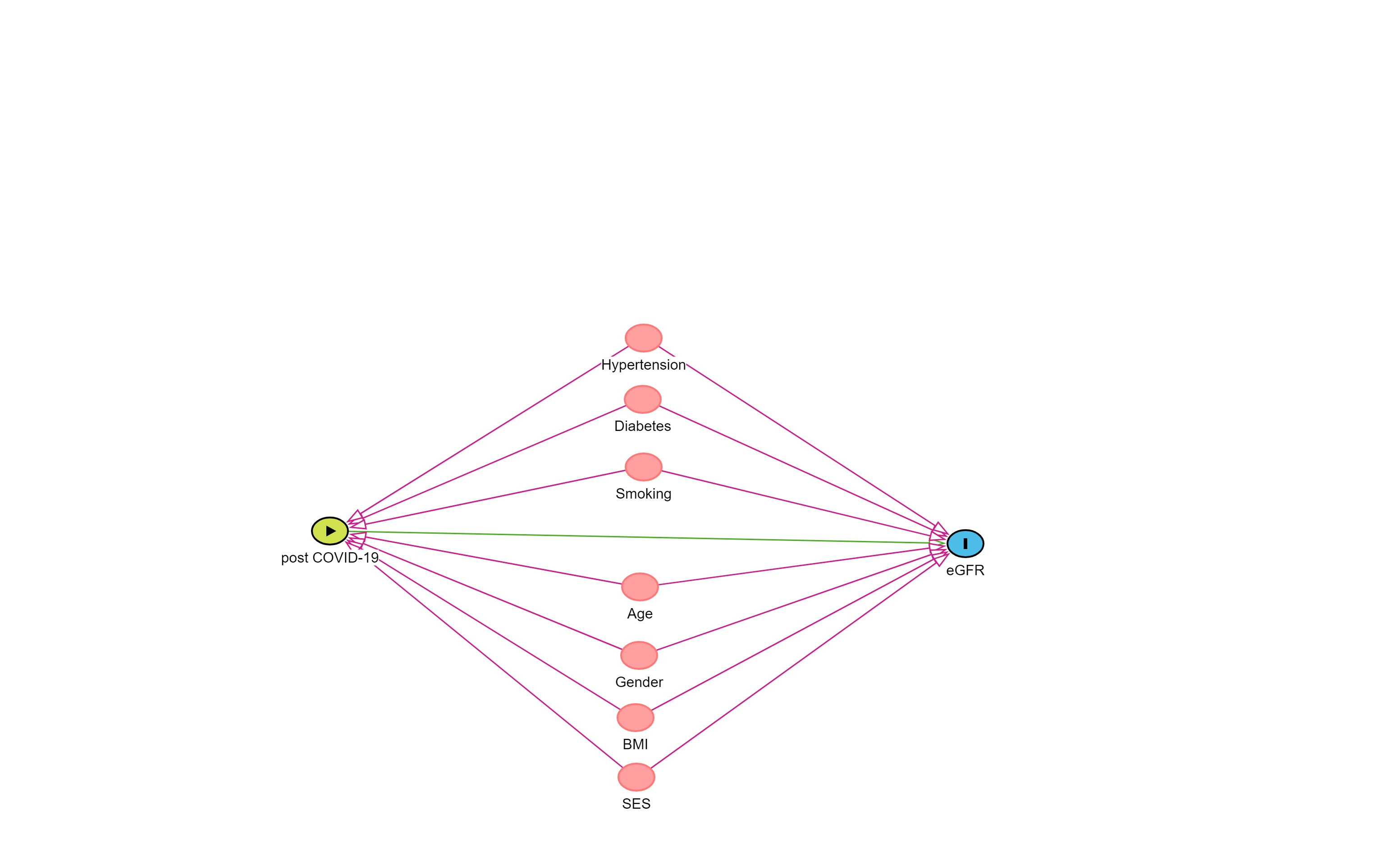


Figure S1. Directed Acyclic Graph of the effect of post COVID-19 on eGFR
The green circle is the exposure (post COVID-19), the blue circle is the outcome (eGFR), and the red circles are the potential confounding factors. BMI: Body Mass Index, eGFR: estimated glomerular filtration rate, SES: social economic status.

Table S1. Sensitivity analysis for the effect of post COVID-19 on eGFR, where controls who never tested positive for COVID-19 were excluded (controls n= 79, post COVID-19 patients n=95)

|  | Estimate (95% CI) | |
| --- | --- | --- |
|  | Unadjusted | Adjusted |
| Post COVID-19 (t=1) | **-8.63 (-13.76, -3.51)** | **-7.69 (-15.07, -0.31)** |
| Post COVID-19 (t=2) | **-9.99 (-15.12, -4.85)** | -5.84 (-11.98, 0.30) |

*Adjusted for age, gender, BMI, smoking, level of education, CVD/hypertension and diabetes.

BMI: body mass index, CVD: cardiovascular disease, CI: confidence interval. Statistically significant results are highlighted in bold (P < 0.05).

Table S2. Sensitivity analysis for the effect of post COVID-19 on eGFR, where all participants with CVD/hypertension were excluded (controls n=90, post COVID-19 patients n=61)

|  | Estimate (95% CI) | |
| --- | --- | --- |
|  | Unadjusted | Adjusted |
| Post COVID-19 (t=1) | **-4.96 (-9.78, -0.13)** | -2.95 (-8.04, 2.14) |
| Post COVID-19 (t=2) | **-8.70 (-14.01, -3.38)** | **-7.02 (-12.82, -1.21)** |

*Adjusted for age, gender, BMI, smoking, level of education and diabetes.

BMI: body mass index, CVD: cardiovascular disease, CI: confidence interval. Statistically significant results are highlighted in bold (P < 0.05).

Table S3. Sensitivity analysis for the effect of post COVID-19 on eGFR, where all participants with comorbidities (CVD/hypertension, diabetes, auto-immune diseases, pulmonary diseases) were excluded (controls n=74, post COVID-19 patients n=36)

|  | Estimate (95% CI) | |
| --- | --- | --- |
|  | Unadjusted | Adjusted |
| Post COVID-19 (t=1) | -2.86 (-8.80, 3.08) | -2.10 (-8.34, 4.15) |
| Post COVID-19 (t=2) | **-9.30 (-16.24, -2.37)** | **-8.80 (-16.22, -1.39)** |

*Adjusted for age, gender, BMI, smoking and level of education.

BMI: body mass index, CVD: cardiovascular disease, CI: confidence interval. Statistically significant results are highlighted in bold (P < 0.05).

Table S4. Sensitivity analysis for the effect of post COVID-19 on eGFR, where post COVID-19 patients aged > 55 years were excluded (controls n=94, post COVID-19 patients n=52)

|  | Estimate (95% CI) | |
| --- | --- | --- |
|  | Unadjusted | Adjusted |
| Post COVID-19 (t=1) | -3.56 (-9.10, 1.99) | -0.70 (-6.51, 5.12) |
| Post COVID-19 (t=2) | **-8.91 (-15.47, -2.34)** | -5.16 (-12.04, 1.72) |

*Adjusted for age, gender, BMI, smoking, level of education, CVD/hypertension and diabetes.

BMI: body mass index, CVD: cardiovascular disease, CI: confidence interval. Statistically significant results are highlighted in bold (P < 0.05).
